# Clinical characteristics and influencing factors of severe fever with thrombocytopenia syndrome complicated by viral myocarditis: a retrospective study

**DOI:** 10.1186/s12879-024-09096-4

**Published:** 2024-02-22

**Authors:** Qian Du, Jin Yu, Qianhui Chen, Xiaoping Chen, Qunqun Jiang, Liping Deng, Anling Li, Yong Xiong

**Affiliations:** 1 Department of Infectious Disease, Zhongnan Hospital of Wuhan University, 430071 Wuhan, China; 2 Department of Neurosurgery, Zhongnan Hospital of Wuhan University, 430071 Wuhan, China; 3 Department of Laboratory Medicine, Zhongnan Hospital of Wuhan University, 430071 Wuhan, China

**Keywords:** Severe fever with thrombocytopenia syndrome, Viral myocarditis, LDH, NT-proBNP, Clinical characteristics

## Abstract

**Objective:**

This study aimed to investigate the clinical characteristics of severe fever with thrombocytopenia syndrome complicated by viral myocarditis (SFTS-VM) and analyze relevant influencing factors.

**Methods:**

Retrospective analysis was conducted on clinical data from 79 SFTS-VM patients, categorized into common (SFTS-CVM, *n* = 40) and severe groups (SFTS-SVM, *n* = 39). Clinical manifestations, laboratory results, cardiac ultrasonography, and electrocardiogram features were analyzed. Univariate and multivariate analyses identified significant indicators, which were further assessed using ROC curves to predict SFTS-SVM.

**Results:**

SFTS-SVM group exhibited higher rates of hypotension, shock, abdominal pain, cough with sputum, and consciousness disorders compared to SFTS-CVM group. Laboratory findings showed elevated platelet count, ALT, AST, amylase, lipase, LDH, D-dimer, procalcitonin, TNI, and NT-proBNP in SFTS-SVM. Abnormal electrocardiograms, especially atrial fibrillation, were more prevalent in SFTS-SVM (*P* < 0.05). Multivariate analysis identified elevated LDH upon admission (OR = 1.004, 95% CI: 1-1.008, *P* = 0.050), elevated NT-proBNP (OR = 1.005, 95% CI: 1.001–1.008, *P* = 0.007), and consciousness disorders (OR = 112.852, 95% CI: 3.676 ~ 3464.292, *P* = 0.007) as independent risk factors for SFTS-SVM. LDH and NT-proBNP had AUCs of 0.728 and 0.744, respectively, in predicting SFTS-SVM. Critical values of LDH (> 978.5U/L) and NT-proBNP (> 857.5pg/ml)) indicated increased likelihood of SFTS progression into SVM.

**Conclusion:**

Elevated LDH, NT-proBNP, and consciousness disorders independently correlate with SFTS-SVM. LDH and NT-proBNP can aid in early identification of SFTS-SVM development when above specified thresholds.

## Introduction

Severe fever with thrombocytopenia syndrome (SFTS) is a zoonotic infectious disease caused by the SFTS virus (SFTSV) infection. SFTSV was first isolated and diagnosed from patient serum by Chinese researchers around 2010 [1, 2]. From then until 2021, a total of 12,953 confirmed cases of SFTS were reported in China, with a high fatality rate ranging from 12 to 30% [3]. Japan and South Korea have also reported local SFTS fatality rates exceeding 20% [4]. Clinical manifestations of SFTS include fever, thrombocytopenia, gastrointestinal symptoms, disseminated intravascular coagulation (DIC), central nervous system symptoms, and multiple organ failure. Additionally, some patients may experience cardiovascular system involvement symptoms such as chest tightness, chest pain, palpitations, and even heart failure. Previous studies have confirmed that SFTSV can directly invade myocardial cells or damage them through secondary immune reactions, ultimately leading to viral myocarditis (VM) [5]. VM is a type of myocardial inflammatory lesion caused by viral infection, which can be classified into common (CVM) and severe (SVM) types based on clinical severity. CVM patients may have no obvious symptoms or only experience discomfort such as palpitations and fatigue, while SVM patients may develop serious clinical complications such as heart failure, malignant arrhythmias, cardiac arrest, and even sudden death [6]. Although there have been few reported cases of SFTS complicated by VM (SFTS-VM), [7–10] systematic and large-sample studies on SFTS-VM are rare. Therefore, this study retrospectively analyzed the clinical characteristics, laboratory indicators, electrocardiogram, and echocardiogram features of 79 SFTS-VM patients, aiming to explore the risk factors for SFTS-SVM. The study intends to provide references for clinical doctors to improve their understanding of SFTS-VM and to identify SFTS-SVM.

## Materials and methods

### Study subjects

**Fig. 1:**
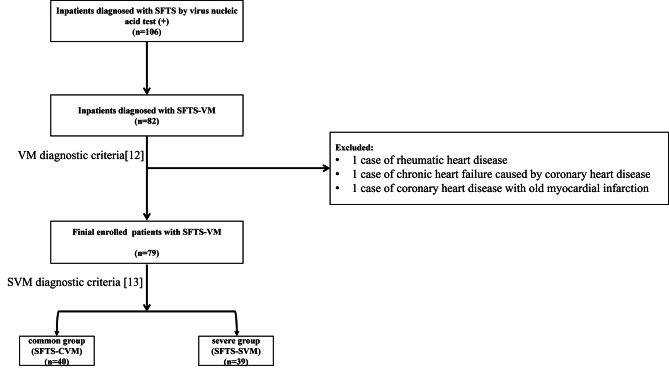
The inclusion and exclusion flow chart of this study. SFTS, severe fever with thrombocytopenia syndrome; SFTS-VM, severe fever with thrombocytopenia syndrome combined with viral myocarditis; SFTS-CVM, severe fever with thrombocytopenia syndrome combined with common viral myocarditis; SFTS-SVM, severe fever with thrombocytopenia syndrome combined with severe viral myocarditis

The inpatients diagnosed with SFTS from the Department of Infectious Diseases in our hospital, admitted from January 2017 to May 2023, were selected as the study subjects. Al l SFTS patients included in this study were diagnosed by positive SFTS virus nucleic acid test through reverse transcription polymerase chain reaction technique from serum samples [11]. All suspected cases were ruled out. Inclusion criteria refer to VM diagnostic criteria of European Society of Cardiology Working Group on Myocardial and Pericardial Diseases [12]. As result, a total of 82 patients with SFTS-VM were preliminarily included. After excluding 1 case of rheumatic heart disease, 1 case of chronic heart failure caused by coronary heart disease, and 1 case of coronary heart disease with old myocardial infarction, a final sample of 79 SFTS-VM patients were enrolled. The diagnostic criteria for SVM referred to the 1999 “Diagnostic Reference Criteria for Adult Acute Viral Myocarditis” and adopted the opinions of the World Health Organization and the International Cardiology Federation Working Group on Myocarditis Definition and Classification [13]. In detail, SVM could be diagnosed if a patient has one or more manifestations of AS syndrome, congestive heart failure with or without myocardial infarct-like ECG changes, cardiogenic shock, acute renal failure, persistent ventricular tachycardia with hypotension, or myocardial pericarditis [13]. Based on these criteria, the SFTS-VM patients were divided into the common group (SFTS-CVM) with 40 cases and the severe group (SFTS-SVM) with 39 cases. (Fig. 1.) This study was approved by the hospital’s ethics committee.

### Data Collection

Clinical data of the included 79 patients, including symptoms, underlying diseases, and complications, as well as laboratory tests (White Blood Cell [WBC], Red Blood Cell [RBC], Hemoglobin [HB], Platelet [PLT], Lymphocyte, Alanine Aminotransferase [ALT], Aspartate Aminotransferase [AST], Albumin, Creatinine, Lactate Dehydrogenase [LDH], Amylase, Lipase, C-Reactive Protein [CRP], Procalcitonin [PCT], D-dimer, Troponin I [TNI], Creatine Kinase-MB [CK-MB], N-terminal pro-B-type Natriuretic Peptide [NT-proBNP] and SFTSV Load) were obtained from our Hospital Information System (HIS). Electrocardiogram findings and echocardiography results were obtained from our Picture Archiving and Communication System (PACS) system.

### Statistical analysis

Data analysis was performed using SPSS 26.0 statistical software. Normally distributed continuous data were expressed as mean ± standard deviation, non-normally distributed continuous data were expressed as median (P25, P75), and categorical data were expressed as percentages (*n*, %). For comparisons between the two groups, independent sample *t*-tests were used for normally distributed continuous data, Mann-Whitney U tests were used for non-normally distributed continuous data, and Pearson’s chi-square tests were used for categorical data. Factors with statistical significance in univariate analysis and those considered clinically or previously significant were included in a binary response variable logistic regression model for multivariate analysis. A *P*-value < 0.05 was considered statistically significant for differences.

## Results

### Basic clinical characteristics comparison

A total of 79 patients with SFTS-VM were included in this study, including 36 males and 43 females, with a mean age of 65.8 ± 7.4 years. 45.6% of patients had underlying diseases, with hypertension being the most common (21%). 53.2% of patients had a clear history of tick exposure upon admission, and 97.5% of patients had combined damage to other organs, with hepatic dysfunction (73.4%) and pancreatitis (68.4%) being the most common. 7.6% of patients eventually progressed to multiple organ dysfunction syndrome (MODS). Among clinical symptoms, 98.7% of patients presented with fever, 17.7% had symptoms of chest tightness or chest pain, and 30% experienced low blood pressure or even shock. Additionally, symptoms of damage to other systems, such as the digestive, respiratory, and central nervous systems, were also observed to varying degrees. In terms of prognosis, 16.5% of patients eventually died. (Table 1)

**Table 1:** Basic clinical characteristics, symptoms, and prognostic analysis of SFTS-VM patients

	Total (*n* = 79)	SFTS-CVM (*n* = 40)	SFTS-SVM (*n* = 39)	*P*-value
**Gender [** ***n*** ** (%)]**				0.73
Male	36 (45.6%)	19 (47.5%)	17 (43.6%)	
Female	43 (54.4%)	21 (52.5%)	22 (56.4%)	
**Age (Mean ± SD) (yrs)**	65.8 ± 7.4	65.0 ± 7.6	66.4 ± 7.3	0.5
**Underlying Diseases [** ***n*** ** (%)]**	36 (45.6)	17 (42.5%)	19 (48.7%)	0.58
Hypertension	21 (26.6%)	11 (27.5%)	10 (25.6%)	0.85
Coronary Heart Disease	7 (8.9%)	2 (5.0%)	5 (12.8%)	0.22
Diabetes	8 (10.1%)	5 (12.5%)	3 (7.7%)	0.48
Chronic Lung Disease	8 (10.1%)	4 (10.0%)	4 (10.3%)	0.97
Chronic Liver Disease	4 (5.1%)	1 (2.5%)	3 (7.7%)	0.29
**Tick Exposure History [** ***n*** ** (%)]**	42 (53.2%)	20 (50.0%)	22 (56.4%)	0.56
**Complications [** ***n*** ** (%)]**	77 (97.5%)	38 (95.0%)	39 (100.0%)	0.16
Hepatic Dysfunction	58 (73.4%)	29 (72.5%)	29 (74.4%)	0.85
Renal Dysfunction	11 (13.9%)	4 (10.0%)	7 (17.9%)	0.31
Pneumonia	47 (59.5%)	18 (45.0%)	29 (74.4%)	0.08
Pancreatitis	54 (68.4%)	29 (72.5%)	25 (64.1%)	0.42
Encephalitis	11 (13.9%)	4 (10.0%)	7 (17.9%)	0.31
DIC	10 (12.7%)	1 (2.5%)	9 (23.1%)	0.06
MODS	6 (7.6%)	1 (2.5%)	5 (12.8%)	0.08
**Symptoms [** ***n*** ** (%)]**				
Fever	78 (98.7%)	40 (100.0%)	38 (97.4%)	0.31
Chills and Rigors	19 (24.1%)	9 (22.5%)	10 (25.6%)	0.74
Muscle Aches	9 (11.4%)	3 (7.5%)	6 (15.4%)	0.27
Headache	28 (35.4%)	16 (40.0%)	12 (30.8%)	0.39
Fatigue	33 (41.8%)	17 (42.5%)	16 (41.0%)	0.89
**Digestive Symptoms [** ***n*** ** (%)]**				
Poor Appetite	18 (22.8%)	8 (20.0%)	10 (25.6%)	0.55
Nausea and Vomiting	32 (40.5%)	16 (40.0%)	16 (41.0%)	0.93
Abdominal Pain	9 (11.4%)	1 (2.5%)	8 (20.5%)	0.01*
Diarrhea	47 (59.5%)	21 (52.5%)	26 (66.7%)	0.2
**Respiratory Symptoms [** ***n*** ** (%)]**				
Cough with Sputum	15 (19.0%)	12 (30.0%)	3 (7.7%)	0.01*
**Central Nervous System Symptoms [** ***n*** ** (%)]**	25 (31.6%)	5 (12.5%)	20 (51.3%)	< 0.01**
**Cardiovascular Symptoms [** ***n*** ** (%)]**				
Chest Tightness or Chest Pain	14 (17.7%)	9 (22.5%)	5 (12.8%)	0.26
Hypotension or Shock	24 (30.4%)	0 (0.0%)	24 (61.5%)	< 0.01**
**Time of Diagnosis Median (P25, P75) (days)**	8 (6–10)	8(6–9)	9(7–10)	0.07
**ICU Monitoring [** ***n*** ** (%)]**				
Yes	26 (32.9%)	5 (12.5%)	21 (53.8%)	< 0.01**
No	53 (67.1%)	35 (87.5%)	18 (46.2%)	
**Prognosis [** ***n*** ** (%)]**				
Death	13 (16.5%)	2 (5.0%)	11 (28.2%)	< 0.05*
Survival	66 (83.5%)	38 (95.0%)	28 (71.8%)	

* *P* < 0.05, ** *P* < 0.01

SFTS-VM, severe fever with thrombocytopenia syndrome combined with viral myocarditis; SFTS-CVM, severe fever with thrombocytopenia syndrome combined with common viral myocarditis; SFTS-SVM, severe fever with thrombocytopenia syndrome combined with severe viral myocarditis; DIC, disseminated intravascular coagulation; MODS, multiple organ dysfunction syndrome

We further divided the SFTS-VM patients into the SFTS-CVM group and the SFTS-SVM group. We found that in terms of clinical manifestations, compared to the SFTS-CVM group, the SFTS-SVM group was more likely to experience abdominal pain (*P* = 0.01), cough with sputum (*P* = 0.01), consciousness disorders (*P* < 0.01), and low blood pressure or even shock (*P* < 0.01). In terms of prognosis, the mortality rate (28.2%) and rate of intensive care unit (ICU) admission (53.8%) were higher in the SFTS-SVM group compared to that (5.0% and 12.5%, respectively) in the SFTS-CVM group (*P* = 0.05 and *P* < 0.01, respectively). There were no statistically significant differences between the two groups in terms of gender, age, underlying diseases, clear epidemiological contact history, and the presence of combined damage to other systems (*P* > 0.05) (Table 1).

### Laboratory examination comparison

In the SFTS-SVM group, the levels of platelets (*P* < 0.05), ALT (*P* = 0.05), AST (*P* < 0.05), amylase (*P* = 0.001), lipase (*P* = 0.001), LDH (*P* < 0.001), D-dimer (*P* = 0.01), and PCT (*P* < 0.001) were all higher than those in the SFTS-CVM group. In terms of myocardial enzyme spectrum, the levels of TNI and NT-proBNP in the SFTS-SVM group were significantly higher than those in the SFTS-CVM group (*P* < 0.001). (Table 2)

**Table 2:** Laboratory examination comparison

	SFTS-CVM	SFTS-SVM	*P*-value
**WBC (10** ^**9**^ **/L)**	2.22 (1.57–3.66)	3.40 (1.65–5.17)	0.16
**Lymphocyte (10** ^**9**^ **/L)**	0.58 (0.43–0.74)	0.51 (0.37–0.87)	0.62
**Platelet (10** ^**9**^ **/L)**	43.50 (35.25-55.00)	37.00 (22.00–49.00)	< 0.05*
**ALT (U/L)**	70.00 (44.75–110.00)	98.00 (65.00-148.00)	< 0.05*
**AST (U/L)**	237.00 (110.00-359.25)	363.00 (222.00-704.00)	< 0.05*
**Creatinine (mmol/L)**	70.95 (64.58–86.35)	71.30 (61.80–98.50)	0.97
**Amylase (U/L)**	128.50 (92.25-177.75)	201.00 (130.00-291.00)	0.001***
**Lipase (U/L)**	135.00 (75.50-264.25)	341.00 (155.00-520.00)	0.001***
**LDH (U/L)**	784.00 (529.50-968.25)	1000.00 (757.00-1451.00)	< 0.001***
**D-dimer (ng/ml)**	1091.00 (397.50-1954.75)	1801.00 (768.00-3468.00)	0.01**
**PCT (ng/ml)**	0.15 (0.05–0.31)	0.44 (0.25–1.04)	< 0.001***
**CRP (mg/L)**	3.35 (2.03–9.83)	5.97 (2.20–11.00)	0.26
**virus loadΔ(copies/ml)**	5965 (438-22950)	24,600 (1080-243000)	0.06
**TNI (pg/ml)**	95.05 (52.40-265.25)	370 (128.10–837.00)	< 0.001***
**CK-MB (U/L)**	39.00 (27.50-58.75)	38.00 (30.00–86.00)	0.51
**NT-proBNP (pg/ml)**	222.50 (76.93–365.50)	967.00 (114.00-3090.00)	< 0.001***

* *P* < 0.05, ** *P* < 0.01, *** *P* < 0.001

SFTS-CVM, severe fever with thrombocytopenia syndrome combined with common viral myocarditis; SFTS-SVM, severe fever with thrombocytopenia syndrome combined with severe viral myocarditis; WBC, White Blood Cell; ALT, Alanine Aminotransferase; AST, Aspartate Aminotransferase; LDH, Lactate Dehydrogenase; PCT, Procalcitonin; CRP, C-Reactive Protein; TNI, Troponin I; CK-MB, Creatine Kinase-MB; NT-proBNP, N-terminal pro-B-type Natriuretic Peptide

Δ The total sample size included in the analysis of this index is 53 patients

### Comparison of electrocardiogram and echocardiogram findings

The proportion of arrhythmias (*P* < 0.004) and atrial fibrillation (*P* = 0.001), was higher in the SFTS-SVM group compared to the SFTS-CVM group, and the differences were statistically significant. However, there were no statistically significant differences (*P* > 0.05) between the two groups in terms of ventricular premature contractions, ventricular tachycardia, atrial premature contractions, atrial tachycardia, ventricular fibrillation, conduction block, ST-T segment changes, ST segment elevation similar to myocardial infarction, abnormal Q waves, low voltage, bradycardia, and QT interval. While in terms of echocardiographic indices, there were no statistically significant differences (*P* > 0.05) between the two groups in ventricular wall thickening, systolic function, diastolic function, cardiac chamber enlargement, valve regurgitation and insufficiency, and pericardial effusion (Table 3).

**Table 3:** Comparison of electrocardiogram and echocardiogram findings

	SFTS-CVM (*n* = 40)	SFTS-SVM (*n* = 39)	*P*-value
**Electrocardiogram**			
No Abnormality	10 (25.00%)	1 (2.56%)	0.004**
Ventricular Premature Beats	1 (2.50%)	2 (5.13%)	0.15
Ventricular Tachycardia	1 (2.50%)	4 (10.26%)	0.16
Atrial Premature Beats	6 (15.00%)	5 (12.82%)	0.78
Atrial Tachycardia	2 (5.00%)	4 (10.26%)	0.38
Ventricular Fibrillation	0 (0.00%)	3 (7.69%)	0.07
Atrial Fibrillation	0 (0.00%)	10 (25.64%)	0.001**
Conduction Block	6 (15.00%)	6 (15.38%)	0.96
ST-T Segment Changes	23 (57.50%)	25 (64.10%)	0.54
ST Segment Elevation Similar to Myocardial Infarction	0 (0.00%)	1 (2.56%)	0.31
Abnormal Q Waves	0 (0.00%)	4 (10.26%)	0.39
Low Voltage	0 (0.00%)	4 (10.26%)	0.39
Bradycardia	5 (12.50%)	3 (7.69%)	0.48
Prolonged QT Interval	3 (7.50%)	3 (7.69%)	0.97
**EchocardiographyΔ**			
No Abnormality	1 (2.56%)	4 (10.26%)	0.35
Ventricular Hypertrophy	2 (5.13%)	3 (7.69%)	0.96
Reduced Systolic Function	1 (2.56%)	3 (7.69%)	0.55
Reduced Diastolic Function	1 (2.56%)	3 (7.69%)	0.55
Cardiac Chamber Enlargement	6 (15.38%)	8 (20.51%)	0.7
Valve Regurgitation and Insufficiency	11 (28.21%)	14 (35.90%)	0.34
Pericardial Effusion	0 (0.00%)	4 (10.26%)	0.09

* *P* < 0.01, ** *P* < 0.01

SFTS-CVM, severe fever with thrombocytopenia syndrome combined with common viral myocarditis; SFTS-SVM, severe fever with thrombocytopenia syndrome combined with severe viral myocarditis;

Δ The total sample size included in the analysis of this examination is 36 patients

### Multiple logistic regression analysis of SFTS-SVM

In the multifactorial analysis, statistically significant factors identified from the univariate analysis and those considered clinically meaningful were included as binary response variables in the logistic regression model, with the presence of SVM as the dependent variable. The results showed that elevated LDH (OR = 1.004, 95% CI: 1.001 to 1.008, *P* = 0.050), elevated NT-proBNP (OR = 1.005, 95% CI: 1.001 to 1.008, *P* = 0.007), and consciousness disorders (OR = 112.852, 95% CI: 3.676 to 3464.292, *P* = 0.007) were associated with an increased risk of developing SVM in patients with SFTS. (Table 4)

**Table 4:** Multiple logistic regression analysis of SFTS-SVM

	OR	95% CI	*P*-value	
**Platelet (10** ^**9**^ **/L)**	1.008	0.956–1.063	0.775	
**ALT (U/L)**	0.997	0.981–1.012	0.658	
**LDH (U/L)**	1.004	1.000–1.008	0.046*	
**Amylase (U/L)**	1.029	1.000–1.058	0.053	
**Lipase (U/L)**	0.994	0.983–1.004	0.253	
**PCT (ng/ml)**	0.579	0.305–1.101	0.096	
**D-dimer (ng/ml)**	1	1.000–1.000	0.252	
**TNI (pg/ml)**	1	0.997–1.003	0.790	
**NT-proBNP (pg/ml)**	1.005	1.001–1.008	0.007**	
**Consciousness Disorder**	112.852	3.676–3464.292	0.007**	
**Abdominal Pain**	370.275	5.58–24570.512	0.06	
**Cough and Sputum**	0.128	0–37.212	0.478	

* *P* < 0.05, ** *P* < 0.01

SFTS-SVM, severe fever with thrombocytopenia syndrome combined with severe viral myocarditis; ALT, Alanine Aminotransferase; LDH, Lactate Dehydrogenase; PCT, Procalcitonin; TNI, Troponin I; NT-proBNP, N-terminal pro-B-type Natriuretic Peptide

**Fig. 2:**
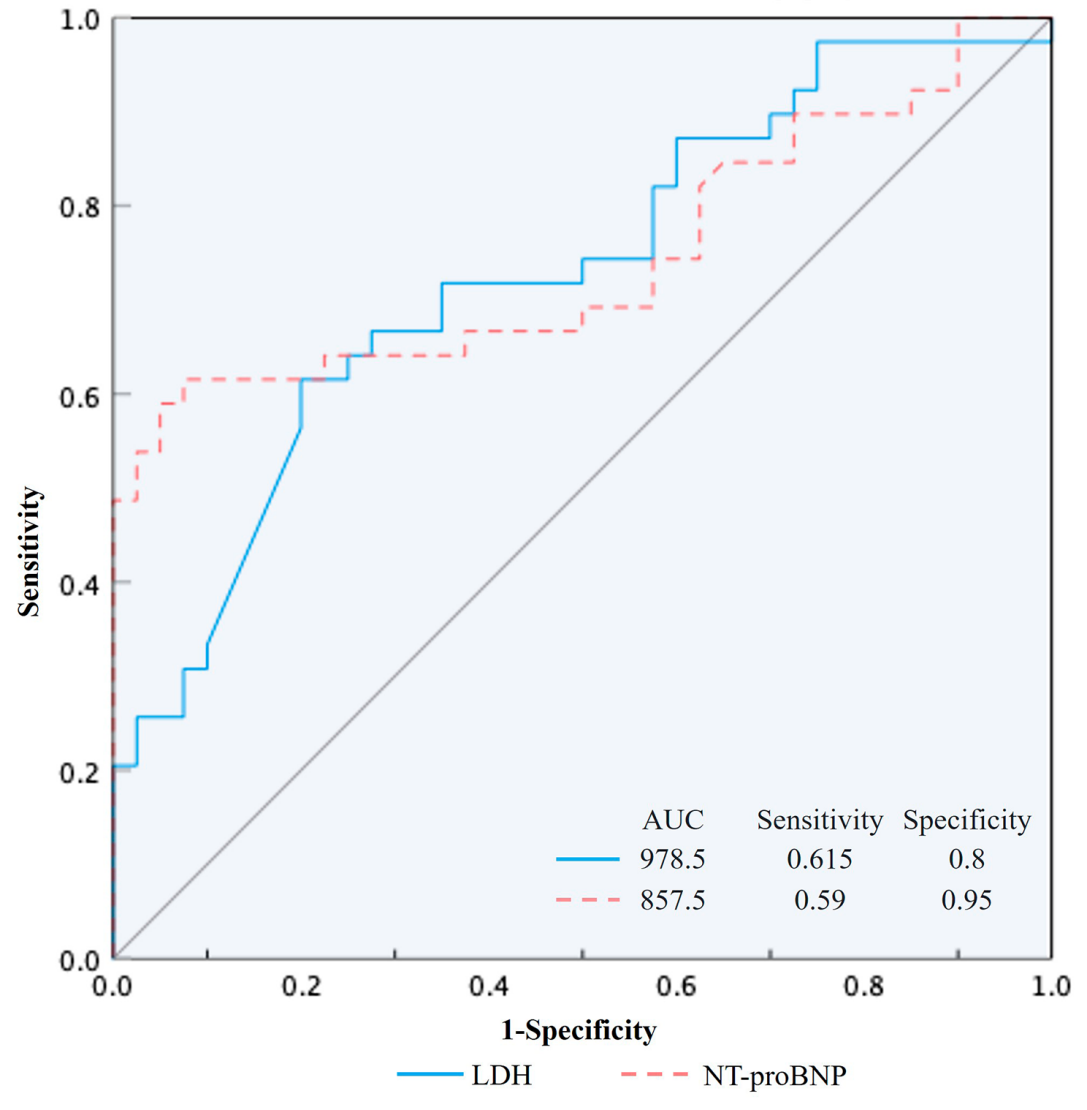
Receiver operating curve (ROC) analysis. The area under the curve (AUC) for lactate dehydrogenase (LDH) and N-terminal pro-B-type natriuretic peptide (NT-proBNP) in predicting severe fever with thrombocytopenia syndrome complicated by severe viral myocarditis (SFTS-SVM) were 0.728 [95% CI (0.616, 0.839)] and 0.744 [95% CI (0.63, 0.858)], respectively. When the critical value for LDH was set at 978.5U/L, the sensitivity and specificity were 61.5% and 80%, respectively. When the critical value for NT-proBNP was set at 857.5pg/ml, the sensitivity and specificity were 59% and 95%, respectively

We further conducted ROC curve analysis on the significant factors identified in the multifactorial analysis. The ROC curve areas for LDH and NT-proBNP in predicting SFTS-SVM were 0.728 [95% CI (0.616, 0.839)] and 0.744 [95% CI (0.63, 0.858)], respectively. When the critical value for LDH was set at 978.5, the sensitivity and specificity were 61.5% and 80%, respectively. When the critical value for NT-proBNP was set at 857.5, the sensitivity and specificity were 59% and 95%, respectively. (Fig. 2)

## Discussion

SFTS is an infectious disease of natural origin primarily transmitted by tick bites carrying the SFTSV. The pathogenesis of SFTS is not yet fully understood, but it is widely believed to involve a “cytokine storm” induced by SFTSV infection, leading to systemic inflammatory response syndrome and ultimately multi-organ damage and organ failure [14]. For example, Yoo JR et al. observed elevated levels of IL-6 and IL-10 in patients with fatal disease compared to those with nonfatal disease at the onset of SFTS. Additionally, the IL-6 and IL-10 levels, rather than viral load and neutralizing antibody titers, strongly influenced the outcomes of SFTSV infection, particularly in cases of severe disease leading to either recovery or death [15]. In 2023, the authors further revealed that the excessive production of IL-10 and IL-6, coupled with reduced TGF-β levels, has been associated with cytokine storm-related fatalities in severe cases of SFTS and critically ill COVID-19 patients. Notably, IL-10 is recognized for its significant role in the host immune response to severe and critical SARS-CoV-2 infections, as well as fatal SFTSV infections [16]. 

Previous studies have detected SFTSV nucleocapsid protein antigens in the liver, spleen, lungs, kidneys, and myocardial cells of deceased SFTS patients, indicating the involvement of multiple organs in this disease [17]. SFTS-VM is one of the complications of SFTS, and its clinical presentation varies. Limited research has been conducted on SFTS-VM, and its underlying causes remain unclear. The occurrence of SFTS-VM is believed to hinge on the direct invasion of cardiomyocytes by SFTSV and subsequent immune responses [18]. Immunohistochemical findings from a severe SFTS case revealed direct SFTSV infection in various organs, including the heart [17].. Additionally, cytokines are implicated in the pathogenesis of SFTS, with elevated levels of IL-6, IL-10, TNF-α, and interferon-γ reported in severe cases, including those involving heart failure, compared to healthy adults [19, 20]. Severe cases may develop malignant arrhythmias, heart failure, or even sudden death. Choi et al. reported that 6.7% of SFTS fatal cases were complicated with myocarditis [21]. In our study, we found a mortality rate of 16.5% in SFTS-VM patients and even 28.2% in SFTS-SVM patients (Table 1). Besides, circulating multimeric immune complexes also contribute to immunopathology in virus infection [22]. Yoo JR et al. found that the titers of neutralizing antibodies, play an important role in protective immunity, to SFTSV in survivors and healthy residents who lived in endemic areas and who were positive for SFTSV IgG, were higher than those in non-survivor patients [23]. Therefore, early recognition and exploration of relevant influencing factors for SFTS-SVM are essential to facilitate early diagnosis, early treatment, and reduce mortality.

The clinical manifestations of SFTS are diverse, with most patients initially presenting with nonspecific symptoms such as fever, fatigue, poor appetite, and muscle aches. As the disease progresses, various systemic manifestations may gradually appear. In our study, we found that 97.5% of SFTS-VM patients had multi-organ damage involving the liver, kidneys, lungs, pancreas, central nervous system, and others. Compared to SFTS-CVM, SFTS-SVM patients were more likely to experience circulatory instability symptoms such as hypotension and shock. Additionally, SFTS-SVM patients had a higher proportion of abdominal pain, cough with sputum, and central nervous system symptoms. These findings suggest that when SFTS-VM patients present with these symptoms, consideration of SFTS-SVM is warranted. The average time from onset to diagnosis for SFTS-VM was 8 days in our study, possibly due to a certain incubation period associated with SFTSV infection. Moreover, approximately 53.5% of patients had a clear history of tick exposure upon admission, indicating the need for vigilance against SFTSV infection when patients present with multi-system symptoms such as abdominal pain, cough with sputum, and central nervous system symptoms in tick-endemic areas. When patients also have symptoms of hypotension and shock, SFTS-SVM should be considered.

Our study found a significant decrease in platelet levels in SFTS-SVM patients. Current research suggests that macrophages in the spleen are the main site of SFTSV replication, and SFTSV can bind to platelets and activate macrophage phagocytosis, leading to reduced platelet count [24]. We also found higher levels of ALT, AST, amylase, lipase, LDH, D-dimer, and procalcitonin in SFTS-SVM patients, which may be related to more frequent occurrences of hepatic dysfunction, pancreatitis, DIC and other SFTS complications with corresponding increases in serum biomarkers [25]. Regarding cardiac enzyme markers, we found a significant elevation of TNI in SFTS-SVM patients, indicating more severe myocardial injury in these patients, while no difference was observed in CK-MB between the two groups, possibly due to the shorter duration of elevation for CK-MB compared to TnI. Because if the time of detection is relatively late, CK-MB values may be in the process of decline. Additionally, we found that approximately 86.1% of SFTS-VM patients had various types of ECG abnormalities, with a higher proportion of arrhythmias, especially atrial fibrillation, in the SFTS-SVM group. VM is characterized by localized or diffuse myocardial edema caused by myocardial cell inflammation, leading to delayed myocardial electrical activity conduction and, therefore, ECG abnormalities [26]. This suggests that ECG examination is essential for SFTS-VM patients, especially when atrial fibrillation is present, as it may indicate the progression to severe disease.

We further conducted multiple-factor logistic regression analysis and found that elevated LDH, NT-proBNP, and consciousness disturbances were associated with an increased risk of SFTS-SVM. LDH is widely present in the heart, liver, skeletal muscles, and kidneys, and is currently considered an early predictive indicator for worsening SFTS and mortality [27]. Our study revealed that elevated LDH is a risk factor for SFTS-SVM, and when LDH > 978.5 U/L, it suggests a greater possibility of progressing to SVM. NT-proBNP is an important serum biomarker for assessing heart function and heart failure severity. Previous studies have demonstrated its predictive value for SVM [28], which is consistent with our study results. SFTS-SVM patients had significantly higher levels of NT-proBNP, indicating a wider range of myocardial injury and poorer heart function. When NT-proBNP > 857.5pg/ml, caution should be taken as it may indicate the development of SFTS-SVM. The presence of consciousness disturbances is also considered a warning sign for worsening SFTS and poor prognosis [29]. It has been reported that SFTSV RNA can be detected in the cerebrospinal fluid of SFTS patients, [30] suggesting invasion of the nervous system and related symptoms. Our study indicates that when SFTS is complicated by consciousness disturbances, patients may be more susceptible to myocardial injury. Considering the above findings, when patients with acute viral myocarditis present with elevated LDH, NT-proBNP, and consciousness disturbances, caution should be exercised as they may be at higher risk of severe viral myocarditis. Early recognition of these patients and proactive life support and anti-inflammatory treatment are essential to improve disease prognosis. If these patients can receive early identification and intervention, their prognosis after the acute phase is expected to be favorable. Caforio AL [7] and Sheng Y [8] respectively reported 2 cases of SFS-SVM patients, both of which showed significant increase of LDH and NT-proBNP in the early stage, and rapidly progressed to severe cardiogenic shock and malignant arrhythmia. Due to the timely diagnosis and identification, these two patients received extracorporeal membrane oxygenation (ECMO) and temporary cardiac pacemaker implantation respectively, and were treated with vasoactive drugs, nutritional myocardium, anti-inflammatory, etc., and finally both patients recovered and were discharged from hospital.

Currently, high levels of SFTSV RNA are generally considered early warning indicators for severe SFTS and poor prognosis [31]. Our study found that the viral RNA level in SFTS-SVM was higher than in SFTS-CVM (*P* = 0.06), but the limited testing conditions only allowed for the detection of viral RNA in some patients, leading to no statistical difference. Future research with larger sample sizes is needed for further study. In addition, we performed echocardiography on some patients and found that about 86.1% of SFTS-VM patients had varying degrees of echocardiographic abnormalities, with valve regurgitation and cardiac chamber enlargement being the most common. However, there was no statistical difference when comparing SFTS-SVM and SFTS-CVM groups, possibly due to the small sample size resulting in bias.

This study has limitations: Most SFTS patients were admitted with severe thrombocytopenia, and considering the risk of coronary angiography and limited significance of cardiac magnetic resonance imaging, patients did not undergo endomyocardial biopsy, coronary angiography, or cardiac magnetic resonance imaging. Therefore, all patients were clinically diagnosed. Additionally, the small sample size may have led to statistical analysis biases. In the future, further verification is necessary through a larger sample size.

In conclusion, our study elucidates the clinical characteristics of patients with SFTS-VM and identifies elevated LDH, NT-proBNP, and consciousness disturbances as independent risk factors for SFTS-SVM. When patients have LDH > 978.5U/L and NT-proBNP > 857.5pg/ml, heightened vigilance should be exercised as they may be at risk of progressing to SFTS-SVM. Early recognition and intervention in SFTS-SVM are crucial to improving prognosis and reducing mortality.

SFTS-VM, severe fever with thrombocytopenia syndrome combined with viral myocarditis; SFTS-CVM, severe fever with thrombocytopenia syndrome combined with common viral myocarditis; SFTS-SVM, severe fever with thrombocytopenia syndrome combined with severe viral myocarditis; DIC, disseminated intravascular coagulation; MODS, multiple organ dysfunction syndrome.

## Data Availability

Data and material are available when asking the corresponding author for information.
